# 3D Printing: Applications in Tissue Engineering, Medical Devices, and Drug Delivery

**DOI:** 10.1208/s12249-022-02242-8

**Published:** 2022-03-17

**Authors:** BG Pavan Kalyan, Lalit Kumar

**Affiliations:** Department of Pharmaceutics, Manipal College of Pharmaceutical Sciences, Manipal Academy of Higher Education, Manipal, Karnataka 576 104 India

**Keywords:** 3-dimensional printing, techniques of 3D printing, tissue engineering, drug designing, disease modelling, medical devices

## Abstract

The gemstone of 3-dimensional (3D) printing shines up from the pyramid of additive manufacturing. Three-dimensional bioprinting technology has been predicted to be a game-changing breakthrough in the pharmaceutical industry since the last decade. It is fast evolving and finds its seats in a variety of domains, including aviation, defense, automobiles, replacement components, architecture, movies, musical instruments, forensic, dentistry, audiology, prosthetics, surgery, food, and fashion industry. In recent years, this miraculous manufacturing technology has become increasingly relevant for pharmaceutical purposes. Computer-aided drug (CAD) model will be developed by computer software and fed into bioprinters. Based on material inputs, the printers will recognize and produce the model scaffold. Techniques including stereolithography, selective laser sintering, selective laser melting, material extrusion, material jetting, inkjet-based, fused deposition modelling, binder deposition, and bioprinting expedite the printing process. Distinct advantages are rapid prototyping, flexible design, print on demand, light and strong parts, fast and cost-effective, and environment friendly. The present review gives a brief description of the conceptional 3-dimensional printing, followed by various techniques involved. A short note was explained about the fabricating materials in the pharmaceutical sector. The beam of light is thrown on the various applications in the pharma and medical arena.

## Introduction

Humans dwell in a computer and technology-driven era. The growth of technology, software, and their advancement is extremely certain in the future. In this fast-changing world, health care is intensifying its stride to adapt new technologies for patient benefit and to venture into uncharted difficult paths. Technology never ceases to astound us with new and unique features that provide simplicity of use and a supplement to the currently existing conventional approach. This incredible technology opens up new possibilities for improving production efficiency in industries.

3D printing is one such astonishing technology that is flourishing in nearly every health care field. They integrate medical and technological applications where the innovation meets the present needs of the health system. 3D printing first popped up in the late 1980s as stereolithography, and it swiftly gained momentum.

**Fig. 1:**
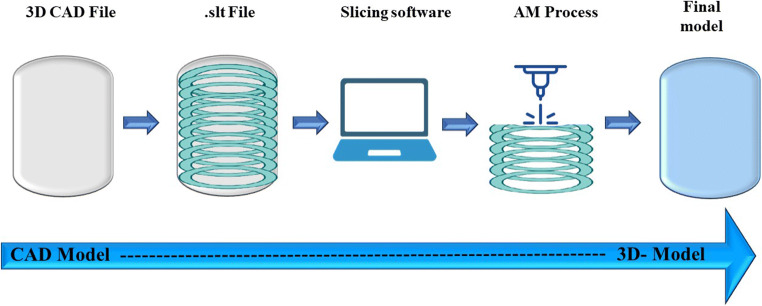
Blueprint of Additive Manufacturing (AM)

This is a miracle technology which utilizes fabrication science discipline with various construction techniques to build various materials of interest (inks) sequentially layer by layer in a range of shapes and forms. It is a straightforward design centered on additive manufacturing (AM) and rapid prototyping (1). Traditional manufacturing sparked the industrialization that took shape to modern society, but it has fundamental flaws that necessitate fresh approaches. AM is a collection of new technologies that build items from the scratch, each cross-sectional layer at a time. This process kicks start the utilization of computer-aided software, generally denoted as CAD, thus creating a 3D model of the desired piece of item. Techno-scientific software slices the provided model in several films, generating the file understandable by AM machine. Thus, the machine imbricates the inks forming the required object of interest (Fig. 1) (2)**.** Selective laser sintering (SLS), stereolithography (SLA), inkjet 3D printing, extrusion, laser-assisted printing, and selective laser melting (SLM) are the few most prominent printing technologies (3).

3D printing has a number of arms to open, expressing its appeals in different sectors counting healthcare industries, such as therapeutics, orthodontics, and medical devices, and the aforementioned innovative technologies are increasingly finding the rise in the clinical study process. Perhaps they spread their wings in other fields including education, apparel, robotics, aerospace, automobile, food, energy, architecture, and cuisine (4). This revolutionary innovation creates a wide range of implantable devices, prostheses, skin engineering, stem line studies, organ modelling, and gadgets for human use which further improvises the outcomes. It adds a beam of hope to pharmacogenetics, personalized medicines, and dosage forms.

Trump cards signifies the major benefits regarding 3D printing are faster, flexible, cost-effective, and customized production, consumer satisfaction, increased quality control, retain time and energy, and better management from collecting raw materials to the final product, rapid prototyping, minimizing the waste, environment friendly which gained an eye in pharma sector (5). Few roadblocks surely peep into this technology as another side of the coin, including many regulatory requirements, stability issue, requirement of suitable bioinks for printing, surface roughness, leaky architectural breakdown, and sterilized production system (6). As there are innumerable benefits implementing and forging the technology ahead, 3D printing is stretching its domain in all medical and non-medical subfields.

This exploration review summarizes the cardinal foundation of 3D printing, technologies based, bioinks utilized for the construction, applications, challenges, and fore-headings of this technology.

## Techniques Behind 3D Printing

The very basic principle supporting 3D printing is additive manufacturing (AM). Unlike conventional machine technology, AM is the process of stacking the connecting materials as layers to give shape as a 3D model. It is a game changer and breakthrough technology which has the potential to lead businesses into a new era of manufacturing and emergence of new business models (7). Few steps are involved in the fabrication process of the desired object. At first, modelling of the object is generally done by use of certain software, preferably computer-aided designing (CAD), generating a high-resolution 3D model. The generated file is saved as Surface Tessellation Language (.stl) configuration, which engenders the object into several triangles. This could be straightforwardly accepted by the printers. As printing is the final stage, the created model is segmented into printable sections. The material of interest is thus stacked constructing the model (8, 9). Every field in which 3D printer technology is applied has gained a new lease of life. It is now feasible to make items that were difficult or impossible to assemble using traditional methods (TM).

The frequently used strategies are aforementioned. The selection of the suitable technique for fabrication depends on the way layers are processed and materials employed (10). Different physical forms of the fabricating materials including solid, liquid, and powder state can be used. Materials embrace are metals, ceramics, and polymers. Metals owe to possess numerous benefits compared to other materials of construction, such as absorption of laser and stability at a greater temperature. Thus, metals are a new bee in this technology where polymers are predominantly used for years (11).

### Fused Deposition Modelling (FDM)

**Fig. 2:**
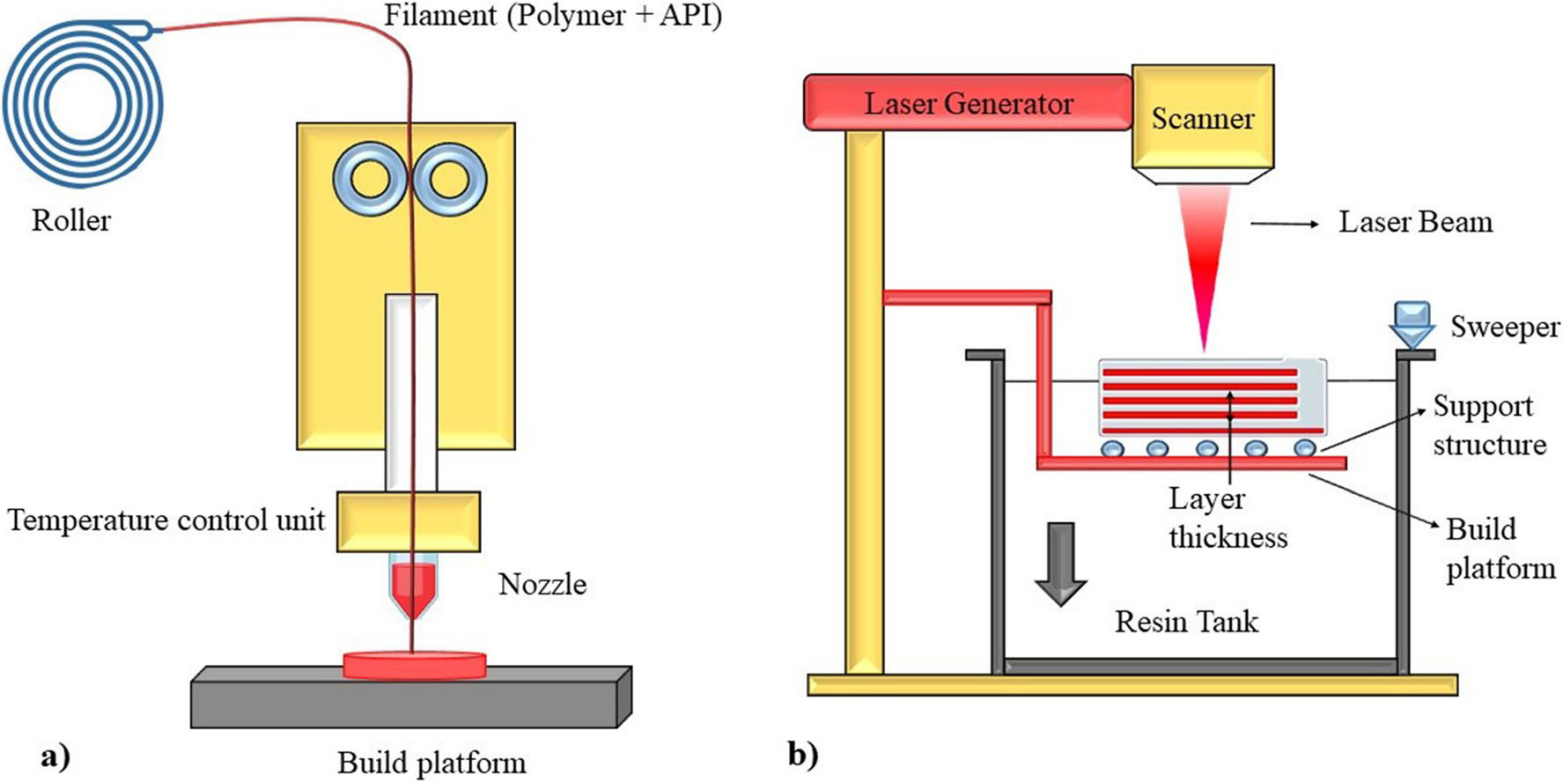
Schematic representation of: **a** Fused Deposition Modelling (FDM), **b** working principle of Stereolithography (SLA)

FDM is an extensively used additive manufacturing (AM), as it possesses some of the industrial benefits for manufacturing. The method comprehends the use of molten thermoplastic polymers (like acrylonitrile butadiene styrene and polylactic acid) which are usually heated up beyond glass transition temperature and sprayed sequentially resulting in layers. The materials keep building of the desired size and layers added are fused together in this way to rise the 3D model (Fig. 2a) (12, 13). The rate of filament given, speed of plotting, and thickness of layer influence the working principle of FDM. Stewart *et al*. (14) formulated an implantable system which aids in the delivery of diverse samples employing 3D printing technology. Based on implant design and sample characteristics, the release rates varied. In addition, a rate-controlling membrane was designed to extend the release indicating its use in treating serious illnesses. Regarding the technique’s simplicity, the results reported that 3D printing is a potential approach for drugs eluting implantation devices (14). Giri *et al*. (15) proposed a unique method for generating gastro-retentive floating tablets. Theophylline was used as a model drug. With zero-order drug release patterns, the result showed sufficient buoyancy for around 10 h. It was proven to be an efficient and cost-effective method for developing gastro-retentive dosage forms with improved controlled release rates (15).

### Stereolithography (SLA)

This technique employs a specialized polymer known as photopolymer, which alters its physicochemical characteristics when UV/IR light hits, via a chemical reaction. It bears some potential supremacy due to its excellent surface texture and quickest and finest resolution 3D printers (Fig. 2b). They bear serious flaws like brittleness, low impact strength, and resistance. Furthermore, they have a constrained life span due to deprivation of physical characteristics with time (16).

Healy *et al*. (17) developed a photopolymer resin formulation by stimulating the SLA method. In this study, aspirin and paracetamol were used as model agents. The researchers examined several polymer ratios as well as two different drug doses. Over the course of the day, medications showed a prolonged release. In addition, attributable to 3D printing, the therapeutic loading capacity and release kinetics were enhanced (17). Xu *et al*. (18) polymerized several antihypertensive drugs as 3D printing dosage form by the aforementioned technique (irbesartan, atenolol, hydrochlorothiazide, and amlodipine). There was a successful polymer topping but a peculiar dark response was reported between photopolymer and drugs. They concluded that there was a need for a better screening procedure to ensure a harmonious drug-polymer relationship (18).

### Selective Laser Sintering (SLS)

**Fig. 3:**
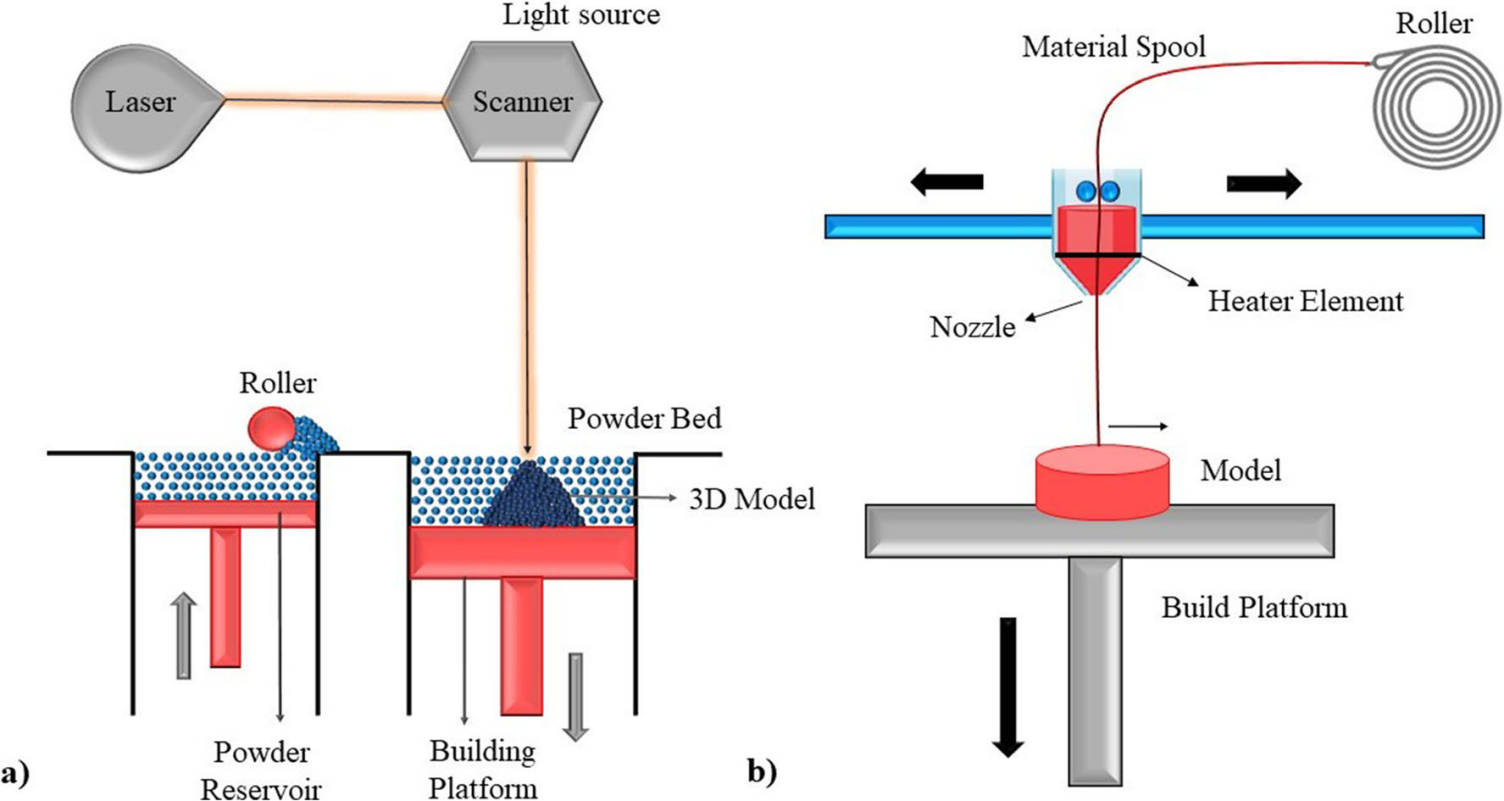
Illustrative scheme of: **a** Selective Laser Sintering (SLS), **b** Material Extrusion

It is a side arm of powder bed fusion (PBF). In this technique, a beam of laser is used to melt and harden photopolymerizable polymer mixture containing therapeutic medication (Fig. 3a). The commercially handy SLS printers feature carbon dioxide (CO_2_) lasers, which emit abundance energy, compatible for the variance range of thermoplastic polymer. The temperature during fabrication, laser intensity, scanning rate, and layer density influences the final requisite model. SLS technology can produce an exorbitant resolution of the model object at room temperature. There seems a curb on the usage of this technique, due to the mutagenic character of the polymer (19). Fina *et al*. (20) presented research that employed SLS technology and followed a single step with elevated resolution. Paracetamol-loaded objects were made out of several polymers. They concluded that this technique may be utilized to personalize therapeutic performance based on the demands of individual patients, with a single standardized ink replacing the necessity for re-designing the formulation mix (20). Thakkar *et al*. (21) utilized SLS to create a three-dimensional dosage form for a light-sensitive therapeutic medication (nifedipine). Visible laser was the source of thermal energy and drugs including nifedipine which undergoes chemical degradation and solid-state transition depending on the various concentrations of the polymers (Kollidon VA 64 and Candurin) was studied. The statistical analysis and design of experiments were performed with a few independent variables (Candurin, temperature at the surface, and momentum of laser). The analysis put forth that Candurin and temperature of the surface were responsible for the negative and positive association on drug degradation and hardness of the generated model respectively. The researchers drew a conclusion that parameters have a relevant footprint on performance and stability when operating the SLS technique (21).

### Selective Laser Melting (SLM)

Selective laser melting (SLM) is also a subclass of powder bed fusion, sharing the domain with SLS. This approach utilizes a bed of powder granules with the specified density. Thermal sources serve as a medium for fusing the particles, as well as a way to regulate the fusion. Laser light projecting on the powder bed generates energy in the form of heat, which melts down the material of construction. The molten mass solidifies further as temperature drops, creating the desired object. Some portion of the bed remains in unmelt form serving as support for the object of interest. Finally, the unused powder bed is removed, once object fabrication ends (22).

### Material Extrusion

Fused deposition modelling is the most well-known example of this technique. This involves several forms of polymers getting deposited, overlapping each other. Specialized movable nozzle glides over the work domain generating a pre-structured model with materials authorized to access (23). Thermoplastic polymers are employed in the extrusion process. Polymers selected are initially heated up to a specific temperature above the glass transition temperature of that polymer. As a result of heating, the polymer melts and forms a layer; subsequently, further layers of polymers are added by extrusion from the nozzle (Fig. 3b) (13). For scaffold construction and hydrogel synthesis, this approach is particularly adaptable and appealing (24).

Burdick and co-workers (25) put forth the crossing nature of a supramolecular network designed as hydrogels. Due to the dynamic properties of the interaction, this extraordinary network boosted the fusion capacity even more. Covalent bonds were responsible for the strong bridging. The robust covalent cross-linking network stabilized the printed structures against the strain of supramolecular network relaxation, which resulted in outstanding printability in multilayered 3D structures printed from dual cross-linked hydrogels. Though the extrusion method possessed a few supremacy conditions like simple and adaptable, it potentially bears some dark sides like requirement of cross-linker, heat energy, and slower rate or printing.

### Inkjet 3D Printing

Inkjet printing allows a specific quantity of items (polymer), typically referred to as ink, to be delivered onto a substrate to build a layer. The appropriate command from computer software kicks starts the printing process and ink ejects out from the printer’s head tank. Depending on ink ejection modes, inkjet printers are classified as continuous mode or drop-on-demand (DOD) mode (26). The continuous ejection system makes use of pressure to initiate the jet form, which is enforced on ink flowing continuously off the printer. Thus, pressurized jet partitions move the stream into precise droplets. DOD printers are further categorized as piezoelectric and thermal printers, relying on the actuator. Following that, an actuator can generate pulses, which results in the ejection of a single droplet with a predetermined ink volume (27).

Walczak and Adamski (28) employed 4 different printers to manufacture microfluidic components using inkjet printing. The minimum size of channels was estimated to be around 200μm. They also argued that removing the support media at the final stage was considered optimum. For characterization, uniform forming dimensions, standard shape, and nature of the outer surface of the modelled products were factored in. The findings suggested that this method might be used instead of the aforementioned rapid prototyping techniques (28).

### Bioprinting

As the nomenclature put forth, bioinks are embedded in this fabrication process. The major domain where this technique finds its appeal is tissue engineering and organogenesis. In particular, spreading the wings in the treatment of fractures and defects associated with bone. Implantation of metal prostheses, grafting of the bones, biomineralization, and osteogenesis seem highly promising using bioink technology. The focus area engaging this method involves the reduction of a few factors including cost, wastage, and time of production in comparison to synthetic methods (29, 30). Categorization of this technique can be extrusion or laser-based methods. The former implies the use of mechanical or pneumatic sources to deposit layers and later represents the laser source which leads to thermal simulation. Kondiah and his colleagues (31) fabricated the bone model by this technology to evaluate the strength, resilience, and porous morphology of the bone matrix. Simvastatin was chosen as the model drug in their research which was incorporated in scaffolds followed by characterization. Optimization formulation demonstration extended therapeutic delivery exceeding 20 days and displayed a generation of pseudo-matrix. The results confirmed that the model’s strength, resilience, and morphology paralleled the characteristics of the human bone (31).

## Fabricating Materials

There are many commercially available polymers, and some models may also demand the use of more than one polymer. The cost of polymer and suitability to the technique used are major hurdles for selection (32). Natural polymers are of particular relevance in 3D printing because of their structural and chemical analogues to the body’s tissue milieu. They also include biocompatibility and biodegradability, both of which contribute to improved biological response. The polymers which are extensively researched are alginate, hyaluronic acid, collagen, and chitosan of natural origin. The major flexibility trump card for synthetic origin includes serene modification to meet physicochemical property, or functionalize diverse biomolecules. Polycarbonate, polylactic acid, polyglycolic acid, and polyethylene glycol are few providing promising advantages (Table I) (33, 34).

**Table I:** Fabricating Materials and Applications in 3D Printing

S. no.	Fabricating materials	Subclass	Examples	Applications
1.	Polymers	Thermoplastics	Polycaprolactone (PCA)	Tissue engineering (Trachea, stem cell model) Drug modelling for cancer therapy
			Poly(lactic-co-glycolic acid) (PLGA)	
			Polylactic acid (PLA)	
			p-hydroxybenzoic acid (PHBA)	
		Thermosets	Urethane Resin	
2.	Hydrogels	Biopolymers	Chitosan	Tissue engineering (bone, cartilage) Drug delivery (nanomedicine)
			Fibrin	
			Collagen	
			Agar	
			Gelatin	
			Alginate	
3.	Composites	Matrix	Carbon fiber	Prostheses, implants
			Silicon carbide	
		Fillers	Hydroxyapatite	
			Calcium phosphates	
			Ceramics precursors	
			Metal precursors	

The most extensively used natural polymer is alginate due to its potential to form hydrogels, shear thinning property, and also exhibiting supreme bioavailability. Duin and his colleagues (35) employed alginate hydrogel to bioprint the functional islets of Langerhans. The pathological feature of type 1 diabetes is the destruction of the insulin-producing pancreatic beta cells. Therefore, a promising strategy to be considered is to control the blood glucose level by transplantation of islets cells. The hydrogel models were employed in encapsulation of the pancreatic cells by applying the technique of extrusion. According to findings, the embedded cells generated insulin and glucagon in a promising manner throughout the study observation (35).

Hyaluronic acid (HA) also comes under natural origin which is found exclusively in the cartilages of bone and other connective tissue systems. Many studies incorporated HA due to its non-adhesivity, and greater capacity of water absorption. Subramaniam *et al*. (36) addressed the periodontitis condition with an application in tissue engineering. Researchers complexed the polymers with calcium sulfate to form a collagenase complex, which served as a substitute for the regeneration of alveolar bone. Micro-CT images were validated to confirm the augmentation of the bone. Herein, researchers reported that the hydroxyapatite-calcium sulfate-hyaluronic acid (HAP/CS/HA) encapsulated collagenase as a novel bone grafting implant for periodontitis therapy, which could promote the restoration of alveolar bone formation without the expense of additional growth factors or cell sources (36).

The prime protein structure that found its way into 3D printing is collagen. The regeneration of rigid connective tissues and cartilage are designed using this proteinaceous polymer. Almeida *et al*. (37) operated 3D printing technology to customize camptothecin loaded in chitosan polymeric micelles. The formulation was further protected from the gastro-intestinal tract by using enteric coating polymer to bypass the harsh environment. The investigation reported that the combination therapy of nanomedicine and 3D printing potentially improved the drug absorption in the intestine, shielded the drug from an acidic environment, and prevented its indignity (37).

On the parallel side, many synthetic polymers also get recognized to be used as fabricating materials for 3D printing technology. Polycaprolactone (PCL) has some dominance in mechanical vigor and processability which enables its suitability for the construction of specific models. Zhang *et al*. (38) proposed a 3D structure wielding magnesium-polycaprolactone loaded with melatonin as a potent antitumor agent. The hypothesis expressed this technology for the treatment of osteosarcoma, bone cancer. The demonstrated investigation analyzed that a 3D-printed drug model could potentially aid in exhibiting the anticancer effect. This model built a novel subservient approach for osteosarcoma treatment (38).

Polylactic acid (PLA) is a fully biodegradable cornstarch-based thermoplastic material. Recent studies proposed that the biocompatibility and mechanical stability of the aforementioned polymer pay the claims in modelling. Asmaria *et al*. (39) aimed to control the likelihood of internal organ failure during the surgery. The generation and validation of the protocol modelling fabricated with PLA were carried out. The qualitative validation technique indicated that there was an enhancement in the modelling ability to envision the existing condition of the patient, while the quantitative technique measured the dimensions of normal and pathological organ prototypes. The result mentioned that the generated gallbladder model mirrored similar settings of biological origin. It concluded that the model amplified the preoperative visualization of the surgery related to the gallbladder (39).

Poly(lactic-co-glycolic acid) is a blend copolymer of polylactic acid (PLA) and polyglycolic acid (PGA). Serris *et al*. (40) conducted a study to fabricate the model drugs paclitaxel and rapamycin with different grades of PLGA in combination or with lidocaine alone. The technique of 3D printing employed was the extrusion-based method. The release profile of the drugs fabricated with PLGA was compared to PLGA-PEG-PLGA hydrogel disks and enhanced release kinetics were expressed by the latter compared to PLGA polymer. The molecular models mimicked the interactions between drug and polymer matrix. The hydrogel disks promised improved release patterns owing to improvement in the profile (40).

Polyethylene glycol (PEG) is a hydrophilic polymer which is generally incorporated in many tissues engineering studies. Singh and Jonnalagadda (41) generated biodegradable mats incorporating the hot-melt extrusion method. The model drug chosen was a topical antibiotic (neomycin) in salt form impregnated with polyethylene glycol and poly-L-lactic acid (PLLA). The mixture of polymers synergistically improved the physicochemical and biological characteristics. Scanning electron microscopy (SEM) showed the uniform particle size and controlled release kinetics for 20 h following the passive diffusion. The reports showed that the threshold was raised when neomycin was built using 3D printing. Furthermore, neomycin-loaded PLLA has its application as a dermal mat and in tissue engineering with the added benefit of PEG polymer boosting its mechanical strength (41).

**Fig. 4:**
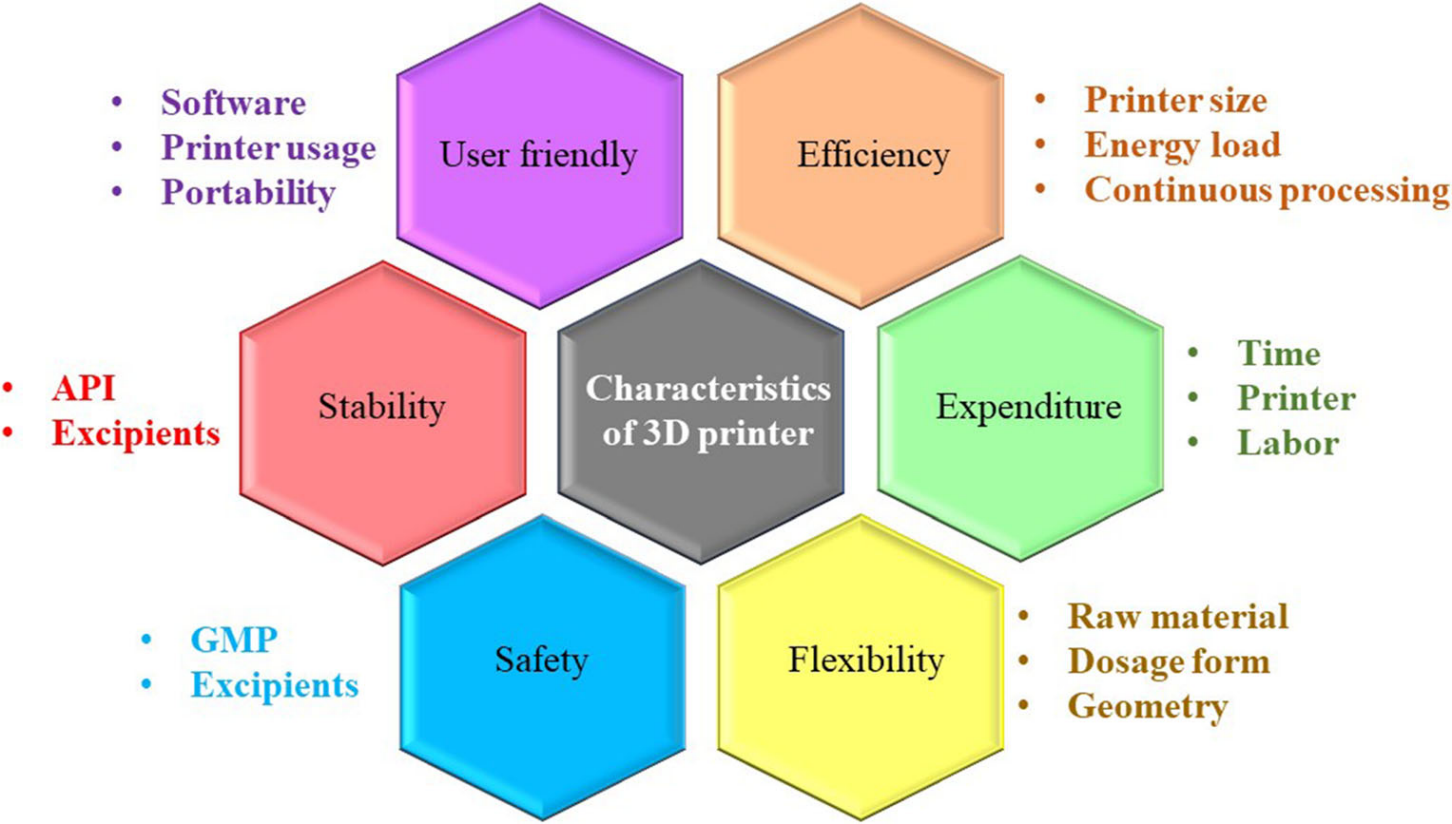
Ideal characteristics of 3D printer

There are different 3D printers which are commercially available, based on the diverse fabricating materials. The selection of the 3D printer sometimes also be based on some ideal characteristics (Fig. 4).

## Applications

**Fig. 5:**
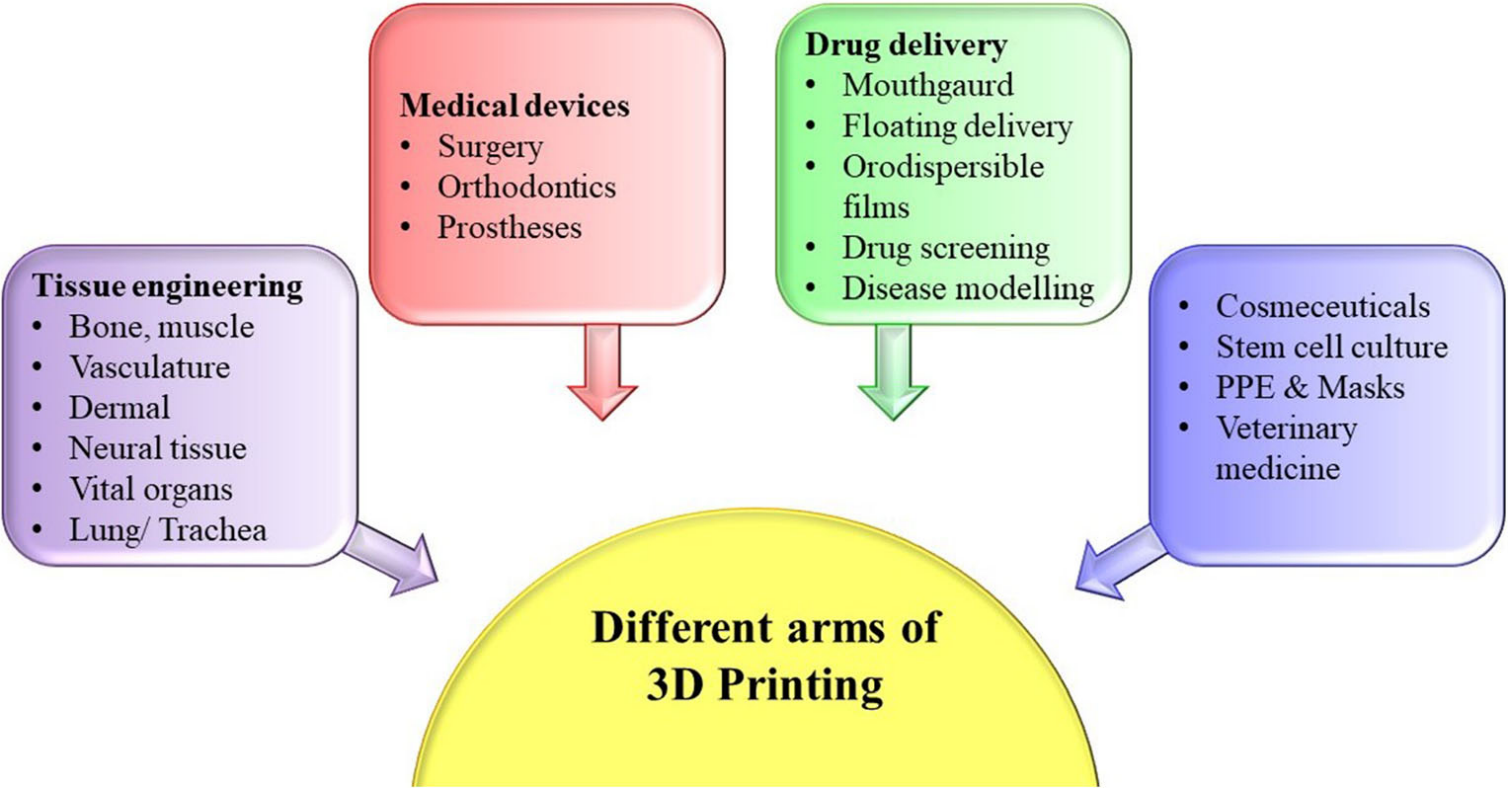
Applications of 3D printing in pharma

The arms of 3D printing are involved in many industrial purposes, among which the ones involved in the pharmaceutical sector are discussed below (Fig. 5).

### Tissue Engineering

**Fig. 6:**
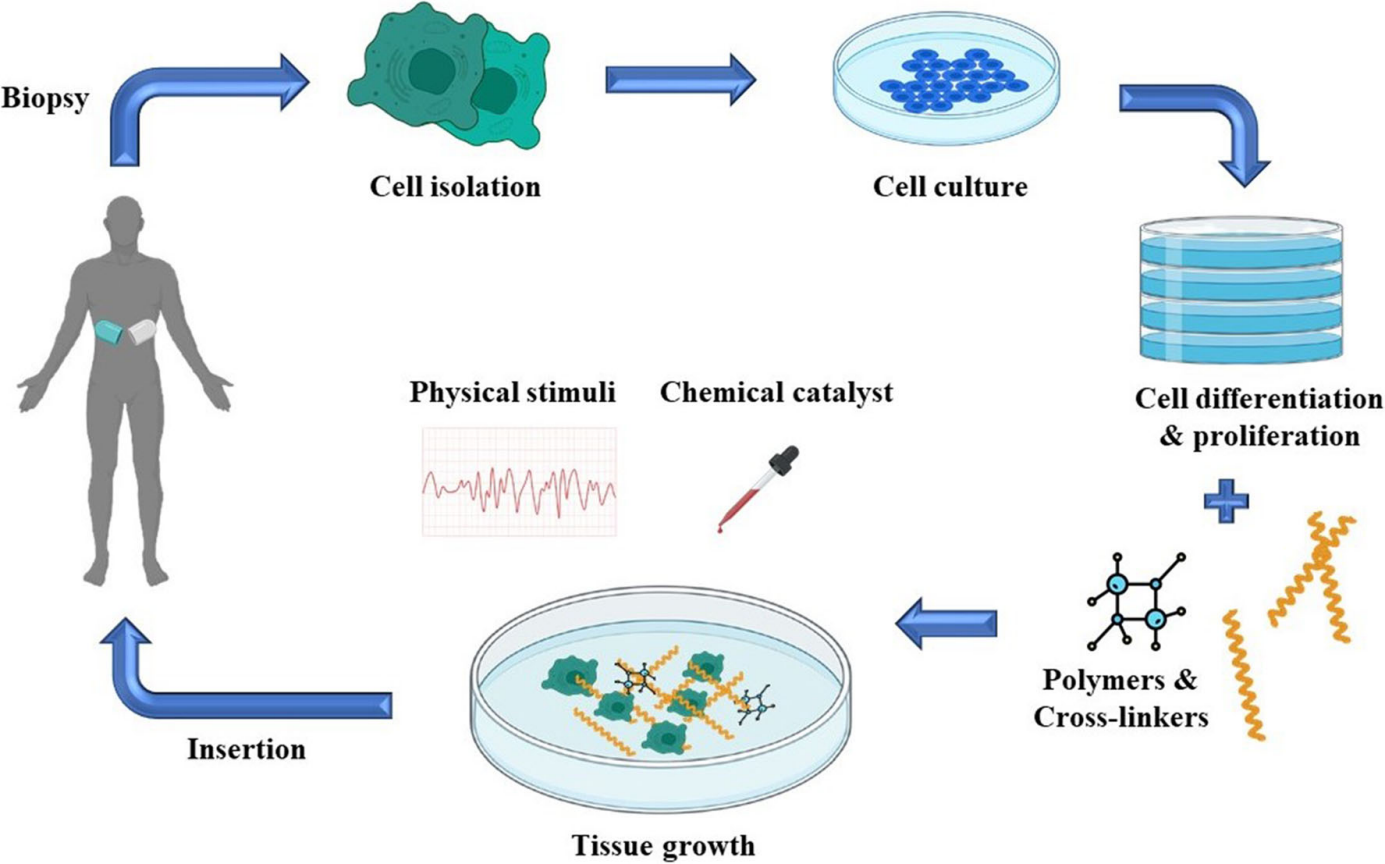
Outline of tissue engineering involving 3D printing

Organ failure is a global concern that has to be addressed. Despite the fact that organ transplantation is an excellent approach for circumventing the failure of a specific organ, it still has a long way to go. The demand for sophisticated technologies is fueled by the unavailability of organs and the long-term necessity for immunosuppressive therapies. As a result, additive manufacturing has made a name for itself in a variety of fabrication tissues, including skin, bone, cartilage, and other vascularized organs like the liver, kidney, and heart, by using bioinks as construction materials (42). Different bioinks find suitable for diverse tissue engineering. Selection of appropriate polymer is a crucial step being the one side of coin, while the characteristics of bioinks also have to be considered. The researcher should have sound knowledge to designate the germane material in the construction (43). These adaptable tissue/organ models have a lot of potential for *in vitro* disease modelling, drug design, high-throughput screening (HTS), and spreading the light on unique platforms such as tissue/organ-on-chip. The generated computer models are instructed into the printer with the polymer materials. According to the predetermined specifications, scaffolds are fabricated (Fig. 6).

#### Bone Tissue

The bone is a hard connective tissue with a dense cross-linked matrix internal structure. Bone regeneration and modelling are required to restore or repair the bone matrix structure and function in a few conditions such as osteoporosis, tumor therapy, and trauma of the bone. The two major focus in bone regeneration is shifted to enhance the ability of regeneration and to mimic the normal conditions of the human bone (44). In comparison, hydrogel-based models are incorporated by many researchers as it possesses easily tunable characteristics. Bai *et al*. (45) focused on hydrogel formulation in the treatment of regeneration of the bone. The appropriate therapeutic outcome was not pleaded when incorporating metal implants or hydroxyapatite as the building materials. Thus, the researchers claimed the unique activity of hydrogels. Further applications of hydrogels in bone regeneration and other tissue engineering methods were expressed (45).

Seok *et al*. (46) fabricated a hydrogel model scaffold with sodium alginate. Though sodium alginate bears retarded binding and poor mechanical firmness, its compatibility and construction efficiency were extra benefits for the formulation model. To overcome the retards, hyaluronic acid was blended with sodium alginate which further improved the compact strength and physicochemical properties of sodium alginate. According to researchers, the scaffold blend of hyaluronic acid and sodium alginate had an effective potency in constructing the bone model paving the approach in tissue engineering (46).

#### Cartilage Regeneration

Cartilage is an elastic tissue which backs the support of many minor organs in the human body. The chondrocytes obtain the nutrients by a diffusion process, as they lack both blood vessels and neurons in their structure. Nguyen *et al*. (47) generated a 3D model of cartilage with the incorporation of two bioink combination namely nanofibrillated cellulose (NFC) with alginate (A) and NFC with hyaluronic acid (HA). Human-derived induced pluripotent stem cells (iPSCs) were the source of modelling. The scholars reported that copolymer of alginate expressed the best pluripotency activity and a further increase in the number of cells improved the density in 3D modelling of the cartilage production (47).

Isaeva *et al*. (48) employed collagen resulting in hydrogel formulation for scaffold model of cartilage. The high concentration of cartilage was incorporated due to its high biocompatibility. Both *in vitro* and *in vivo* characteristics were studied by scholars to check the suitability of collagen polymer. The former study suggested that cells did not possess the capability to survive in specific conditions while weak inflammation followed by the formation of connective fibers and macrophages around the nucleus was expressed in the latter study. Thus, they concluded that the number of chondrocytes formed was insufficient to commence cartilage tissue growth (48).

#### Skeletal Muscle Regeneration

Myofibril is the functional unit constituting the skeletal muscle structure. The engineering of this organized layered structure was made possible by 3D printing. Merceron *et al*. (49) integrated 3D printing for the fabrication of skeletal muscle which possesses varying mechanical strength and cell kinds. In this study, four different polymers were utilized to construct the tendon-muscle model. On one side, polyurethane was printed alongside a hydrogel-based polymer owing to the development and flexible nature of the muscle. On the other side, polycaprolactone was blended with a similar hydrogel formulation which promotes the stiffness and elongation of tendons. With respect to specific mechanical and biological characteristics, the fabricated model satisfied the purpose facilitated by tissue engineering (49).

Dickman *et al*. (50) explored the model of muscular contraction, which was a major stumbling block in a number of diseases such as asthma and bowel illness. They demonstrated a 3D model of muscle for bypassing this difficulty. The bioink used was collagen polymer. The model was fabricated which mimicked the respiratory pathway and muscle associated with it. The generated 3D model was constricted and eased by the act of drugs like histamine and salbutamol, respectively. In the period of a day, the contraction of muscle was observed and the degree of contraction grew dramatically as the number of days increased. This contraction mechanism was particularly specific in matured muscles that were agonist dependent. To generate and sustain long-term alterations in muscle movements, fibrosis was promoted. As a result, the fabricated model demonstrated a promising resemblance to biological muscle and served as a platform for delivering drugs to the muscle (50).

#### Vascular Regeneration

Blood vessels play a crucial role in the transportation of nutrients, and gases like oxygen for various internal organs. Blood is the connective tissue which is very much essential for the functioning of the organs. In many of the trauma and diseased conditions, major vasculatures are damaged or interrupted making it difficult to exchange gases and nutrients among the organs. The 3D printing technology puts a ray of hope in the regeneration of the vessels as tissue engineering became easier. Jin *et al*. (51) advanced the tissue engineering technology in the regeneration of the blood vessels in addition to muscle and endothelial cells. Polycaprolactone (PCL) and methacrylate gelatin were the major polymers employed. The electrospinning method was employed to PCL in order to improve the adhesion property and also functionalization possessing elastic characteristics. Furthermore, the framework allowed the gelatin polymer to be organized in a linear pattern with the muscle cells using a rotatory bioprinter. The aforementioned construction model synergistically added the benefits of two technologies and served as a new strategy for modelling the vascular tissues (51).

Hann *et al*. (52) integrated two fabrication techniques for the development of a vascularized bone tissue model employing tissue engineering. Bone illness and deformities rose due to many reasons including trauma and accidents. So, the application of 3D printing in tissue engineering is a key boon to model the structures with the reaping benefits. In this study, a synergistic combination of SLA and FDM was put forward to successfully model the bone with the vasculature. Firstly, the scaffold hallow model of polyvinyl alcohol (PVA) was fabricated. Inside the core, bone tissue was generated via the proliferation of mesenchymal cells of human bone marrow, and for the capillaries, endothelial cells from the umbilical vein were incorporated into the structure. This unique approach satisfied the generation of bone tissue with vasculature mimicking the typical human blood capillaries (52).

#### Dermal Regeneration

The skin comprising the epidermis (outermost later), dermis (vascularized layer), and hypodermis (adipose tissue) covers the major surface over the human body. The skin being the largest organ in the human body generally provides protection against mechanical shock and maintains thermal homeostasis (53). Ma *et al*. (54) expressed the major back holds for skin regeneration permanently. Improper vascular network, insufficient angiogenesis stimulation, and regeneration of skin were the challenging factors. As researchers were primarily focused on the induction of angiogenesis, the microstructures of strontium silicate were generated and embedded in the bioink polymer. The cell writing 3D printing technique was employed to model the vascularized tissue which exhibited the angiogenic activity in both *in vitro* and *in vivo* circumstances. This research provided the path for tissue engineering of vascularized skin which has a faster recovery rate (54).

Another piece of work in regeneration of the skin generating the scaffold in structure was investigated by the scientists Afghah and his colleagues. (55). The fabricated scaffold in structure proved to be biodegradable, and biocompatible. As silver particles have antibacterial effects, they were combined with a co-mixture of two polymers, namely polycaprolactone and propylene succinate. The hydrolysis and enzymatic breakdown tendency were enhanced by combining suitable polymers, in addition to other significant properties. Silver particles expressed the suppression of proliferation of specific microorganisms invading the model structure which added a major benefit. As a result, researchers concluded that using 3D printing to compute enhanced degradation behavior and antimicrobial activity, which remunerated the path for tissue engineering and wound healing (55).

#### Neural Tissue Regeneration

Neurons are an integral part of the nervous system. These structures carry information in the form of electrical impulses between different tissues and organs. Many neurodegenerative disorders rise away in this era, limiting the autonomous regeneration of the defective neurons. 3D printing technology addressed the advanced breakthroughs in this arena. Liu *et al*. (56) addressed an end to peripheral nerve injury by embedding the combination of 3D printing technologies. This study designed and fabricated the tunable nerve tissue which bears similar biological characteristics. Electrohydrodynamic (EHD), dip printing, jet printing, and electrospinning techniques were used in an empirical sequence in modelling the tissue. Firstly, the core polycaprolactone (PCL) filaments were printed by EHD, the intermediate layer of gelatin in hydrogel form by dip technique, and finally the formed layers were spun into polycaprolactone nanofibers. With the ease and potentiation of modelling methods, the results exhibited the desired tunable characteristics and resembled the normal human neuron (56). Ye *et al*. (57) used a biodegradable polymer, gelatin methacrylate in hydrogel form, to regenerate the peripheral nerve cell. Employing digital light processing, this fabrication model fixed the large gap nerve injury. The supplementary multichannel tunnels were simulated, and co-culturing with PC12 cells revealed the improved neural cell proliferation, design, and transit via the microchannels. In the modelling of neurons which mimics the original human architecture, this technique was known to be efficient and adaptable (57).

#### Spinal Cord Regeneration

The spinal cord is the pathway of transmission of information to and from the brain to different other organs of the body. Thus, efferent and afferent neurons play the role of carrying the various stimuli accordingly. Trauma of the spinal cord is a threatening alarm as most of the information finds the roadblock before reaching the brain cells, as the spinal cord bears many different cell types in highly spatial distribution. This contributed to the poor diagnosis functions and repairability for many of the spinal cord conditions. The advancement of tissue engineering by 3D printing addressed this issue in a great effective and reproducible manner. The bioprinting method allows the growth and development of cells inside the predetermined scaffold structure. Liu *et al*. (58) put forth the importance of 3D printing technology (extrusion) maintained under low-temperature conditions. The fundamental advantage of low temperatures was that they allowed the biological cells to proliferate with a variety of growth factors, while also maintaining their biological characteristics. The researchers introduced a framework that included brain-derived neurotropic factors incorporated in collagen or chitosan polymers. The release of neurotropic factors was prolonged using this low-temperature fabrication approach, which aided in the regeneration of spinal tissues as a treatment for traumatic spinal disorders. Improvement of locomotory functioning by regeneration, scar reduction, and cavity development was observed in the experimental animals. They concluded that this modelling could potentially be utilized in the construction and development of the spine network (58).

#### Cardiac Tissue Regeneration

The circulatory system carries numerous nutrients, gases, and xenobiotics to all of the organs in the human body. Some cardiac and circulation irregularities lead to a variety of disorders, many of them being fatal. As the cardiovascular system is made of a variety of tissues, transplantation may be the best option for treatment. The hurdle in transplantation was the availability of minimal donors with very few biological constraints associated with it. To address this, a great leap was established by 3D printing to fabricate the specific tissues with the suitable biological matter in it, which owes to improve the transplantation meeting the demands and also compatibility with the biological system (59). Though 3D printing technology could be used in heart tissue regeneration, mimicking the exact biological action was a back hold in the technology. As the technology uses patient-specific cells, organ rejection was greatly controlled. 3D printing and bioprinting technologies have already been extensively included in many of the investigations for the treatment of congenital cardiac disease, aortic aneurysms, cardiac tumors, and other cardiovascular disorders (60–62).

Lee and his associates (63) employed collagen to generate cardiac tissues that mimicked the physiological process. The novel method of suspended hydrogel form was engineered to scale up the capillaries to complete organs since the scaffold model of collagen was difficult to functionalize the biological tissue. For the manufacturing process, the porous nature of the model imparted rigidity and microvascularization. Cardiomyocytes were employed in the production of tissue in the scaffold hydrogel. The proposed model expressed the synchronized contractions, and directional action potential transmission as the potential way for the modelling of the cardiac tissues (63). Zhang *et al*. (64) fabricated the myocardium with the application of 3D printing. Bioinks were used to facilitate the model which resulted in the scaffold structure. The endothelial cells were imprinted into the microfiber layers constituting the model. Furthermore, the cardiomyocytes were obtained from the pluripotent stem cells and seeded with endothelial cells in the structure. Researchers thus embedded the organoids into a specially designed microfluidic perfusion bioreactor which completed the whole organ on chip model. This innovative model accurately imitated the human cardiomyocytes, paving the possibility for regenerative medicine (64).

#### Liver Regeneration

The liver is the largest important internal organ in the human body, since it is responsible for the metabolism of the majority of endogenous and exogenous chemicals that enter the body. 3D printing technique is used for disease modelling, pharmaceutical screening, and organogenesis. Yang *et al*. (65) investigated hepatic modelling and its application in the treatment of end-stage liver failure. The production of hepatocytes by the use of specialized bioinks was made possible because of 3D printing technology. After a week of cell proliferation in a controlled *in vitro* settings, the cells were implanted into Fah-deficient animals (mice). Survival time and liver damage in mice, human liver function indicators, and particular metabolite synthesis were the main observations made on 3D-printed hepatorganoids. According to the conclusions, specific hepatic activities such as albumin, glycogen secretion, and drug metabolism were evaluated and reported the improved properties. As a result, the developed model had the ability to alternate organ transplantation from donor bodies (65). Mao *et al*. (66) created liver microtissues by refining the digital light technique, which aids in the treatment of end-stage liver illness. Microtissue was synthesized and tailored to improve liver function. The bioinks that are specific in fabricating the liver were employed. The blends of methacrylate gelatin along with the extracellular matrix were developed. The human-induced hepatocytes were incorporated into the model outline under specific conditions. Several parameters were assessed, including swelling property, mechanical strength, and polymer compatibility. In the simulated 3D model, an increased albumin secretion and urea excretion indicated hepatocyte growth. As a function, the model produced was biosimilar to the biological origin of the transplant (66).

#### Islet Regeneration

Type 1 diabetes mellitus is an autoimmune disorder wherein the pancreas produces negligible or no insulin. The immune system assaults the insulin-secreting beta cells in the pancreas and also the lack of blood arteries made a challenging modelling technique. Despite the fact that insulin supplements are most often utilized, the pancreatic islet transplantation provides a long-term disease management. The progress of 3D printing using hydrogel polymer composition was designed and refined to fulfil the demands of patients. Lui and his colleagues (67) built a scaffold using biodegradable polymers such as alginate and gelatin-based hydrogels. The coaxial printer was used to customize the multicellular islets. The islets cells were characterized, and their vitality was maintained throughout the modelling process. The cells in the scaffold structure resembled the proliferation of normal human cells which needs further investigation for the compatibility in transplantation (67).

#### Trachea Regeneration

The trachea is the organ responsible for transporting gases to the bronchi and lungs. In many cases, the trachea is affected, which has an influence on lung capacity and function. There occurs a technology that can adjust the functionality by regenerating the trachea, mimicking the human trachea. The incorporation and proliferation of normal vasculature in the model structure were a stumbling block, which was further simplified by 3D printing (68). Park *et al*. (69) aimed to design a scaffold model for the regeneration of the trachea by using 3D printing. The core model of the trachea was made of polycaprolactone and hydrogel, with endothelial cells from the nasal cavity and cartilage tissues added for proliferation. *In vivo* assessment of the simulated scaffold constructions in experimental animals (rabbits) was examined. The animals were randomized into two groups: control and experimental. The control group bore six animals, whereas the experimental group bore fifteen. The difference between groups was that the scaffold model lacked endothelial cells and cartilage in the control group. According to the results of *in vivo* trials, three out of six rats in the control group perished owing to respiratory complications. On the other hand, the proliferation of endothelial cells was seen in the experimental group animals. However, there was no evidence of cartilage formation (69).

Kim *et al*. (70) developed an artificial trachea model that was biosimilar to the natural human system in terms of characteristics and functionality. On the polymeric scaffold framework, the researchers presented two folds of tubular and pluripotent stem cells. The biopolymer polycaprolactone was employed as the two structurally different forms: nanofibers of the polymer as the inner core and microfibers as the outer surface. To ameliorate the tracheal mucosa and cartilage, epithelial cells of bronchi, mesenchymal cells, and chondrocytes were used. For *in vivo* assessment trials, rabbit models were selected. Experimental animals were given a tracheal model, and the findings were examined. Micro-computed tomography described the formation of cartilage in the damaged part of the trachea, whereas Alcian blue staining revealed the proliferation of columnar epithelial cells. This study demonstrated to be a potential method for regenerating segmental trachea defects (70).

#### Lung Regeneration

Due to limited donor organs and immune system rejection, lung transplantation poses a serious concern. The transplantation proved impracticable and required a lot of constraints due to a few drawbacks. Lung tissue engineering offers a solution to the problem of lung transplantation shortages. The growth of 3D printing conjunction with normal cells is a potential method in the development of lung tissue engineering (71). The nanofibers were synthesized by Huang *et al*. (72), using 3D printing of unique bioinks in the design process. Hydrogen peroxide was cross-linked with silk fibroin, and oxidized bacterial cellulose was supposed to improve the scaffold model's structural qualities by increasing the viscosity. The hydrogel was coated over 10 times to increase the model's durability as well as rigidity. Under this study’s design, several particular parameters such as printing pressure, printing speed, and nozzle diameter were also optimized. When nanofibers were retained in the culture for seven days, they proved to aid in the orientation of the lung epithelial cells as well as their further proliferation. According to the researchers, nanofibers formed from the aforementioned polymers might be a potential tissue engineering technique for lung regeneration (72).

### Medical Devices

Medical devices play a significant part in how we are moving towards innovation. They find their applications for specially abled persons and also seek a role in surgery, orthodontics, and prosthetics. Thus, 3D printing technology paves a greater way in the generation of personalized devices in the field of medical sciences. It may also be feasible to develop devices that would be difficult to make using conventional approaches. Food and Drug Administration (FDA) granted the market access for a few medical devices fabricated employing 3D printing to promote its use among the individuals. Few devices are enlisted in Table II (73).

**Table II:** Few FDA-Approved Medical Devices Employing 3D Printing

Medical device	Medical application	Description
Lateral spine truss system	Spine	It acts as a framework for cell adhesion and bone growth. The open architecture enables bone development. The implant’s bi-convex shape pushes it nearest to the neighboring bone.
HAWKEYE vertebral body replacement system	Spine	To replace a collapsed, injured, or unstable vertebral body owing to malignancy or trauma in thoracolumbar and cervical spines.
Cellular titanium cervical cage	Spine	Inserted using an anterior technique to restore intervertebral length and assist body integration in the cervical spine
Unite 3D bridge fixation system	Foot and ankle joint	In the midfoot and hindfoot, it provides a solid and long-lasting solution for fracture and osteotomy repair, as well as joint arthrodesis.
ELEOS limb salvage system	Oncology	Surgeries requiring radical resection and replacement of the distal femur, proximal femur, proximal tibia, or entire femur, especially in cancer stages.
Teeth aligners (candid)	Orthodontics	Personalized teeth braces for the individuals
Virto B-titanium hearing aids	Hearing aid	Personalized hearing aids automatically adjustable to the surroundings with excellent performance

#### Orthodontics

Three-dimensional medical imaging enables more accurate diagnosis and production of orthodontic equipment based on the occlusal plane’s coordinate system inside the dental anatomy. With breakthroughs in medical imaging, 3D printing, and customization of appliances and devices, orthodontics and dentistry are witnessing a technological revolution (74). Therefore, this technology cleared the vision of fabricating the dental models with a variety of materials, enhanced comfortability, and reduced treatment period. Redaelli *et al*. (75) evaluated the feasibility of the orthodontics braces by 3D printing. The polymer taken into fabrication technique was selected as polyethylene terephthalate glycol–modified. The printed model was thus compared with thermoformed polymers including polyethylene and polypropylene. The parameters evaluated for the study were tensile characteristics, affordability, mechanical strength, cost, and acceptability of the prostheses on human patients. Results demonstrated that the proposed technique meets the present need of the patient. The structural model had no flaws in terms of morphological properties, and mechanical strength was determined to be increased with improved patient compliance. To demonstrate pharmacological action, this customizable model can also include therapeutic drugs (75). Thurzo *et al*. (76) utilized 3D printing to create the model for the orthodontic power arm. The biocompatible polymers were used in the model’s construction. The porous alloy type of titanium was ideal for biomedical applications. For the enhancement of power-arm robustness, the researchers used finite element modelling. The model was attached to tooth contour and then the studied design was able to construct. The construction culminated in a 7 percent increase in tensile arm strength and an 82 percent reduction in stress, demonstrating the enhanced qualities over standard technique (76).

#### Prothesis

Researchers and clinicians are capable of building totally user-friendly prostheses with the help of 3D-printable devices, which are transforming the face of medicine. Developing flexible, high-quality prostheses, and more comforts for individuals is a challenge faced globally, since such devices are costly and also leads to user discomfort. Prosthetists can benefit greatly from new technology like 3D printing, which aids in promoting the innovation of personalized prostheses (77).

In order to reduce the risk of bacterial infection following amputation, Zuniga (78) proposed a solution to this vexing challenge. Plactive (3D printing filament) with antibacterial properties was employed in this study. The goal was to emphasize the use of antibacterial materials in this model’s production and further characterization for therapeutic properties. For the finger amputation experiment, two adults were included; a 3D-printed finger prosthesis was attached to the amputated part. The antibacterial activity was tested against a few topical microorganisms and indicated that 99.99 percent of antibacterial nanoparticles were effective. This innovation leads to patient satisfaction and comfort towards the modelled prosthetics (78).

Honigmann *et al*. (79) conducted a study with the goal of scaffolding scaphoid prosthesis using a desktop 3D printer. The model was integrated employing the fused filament technique. The building biomaterial, polyetheretherketone (PEEK), was chosen as a medical-grade material. PEEK offered the convenience of reducing the risk of particle-induced implant loosening while also limiting metal-metal debris. The assembly of class II biomedicals was aided by this 3D model. As a personalized product, this model drew a lot of attention. A comparison was done between biopolymers of medical and industrial standards. To summarize, this prosthetics model demonstrated improved integration and efficient PEEK production (79).

Xiao *et al*. (80) compared 3D model prostheses to traditional prostheses. The modelling process combined stereolithography and physical vapor deposition techniques. The construction material was titanium-coated polymer, demonstrating its benefits in medical and biological domains. The compression and crushing strength of the mandibular model were investigated. The outcome was that the titanium model could endure a compression force of more than 20% while also having increased compression strength. Researchers predicted the finite element analysis to enhance the design and mechanical and biological aspects in the near future (80).

#### Surgery

Surgery is a complicated tremendous process which generally involves many complex complications. By the inclusion of advanced technologies, the risk during and postoperative conditions was cut down. The overarching objective is to reduce the operation time and risk involved, while also improving compliance and quality. Thus, 3D printing lays a platform for improved surgical outcomes in difficult scenarios. The emergence of this technology piqued the curiosity of academics and surgeons, routing its applications in various fields (81).

Chen *et al*. (82) adopted 3D printing in the construction of the mesenteric vessels in right hemi-colon cancer surgery. The randomized clinical studies were performed with 3 different groups as 3D printing, 3D image, and control divisions. The key data were operation time, volume of blood bleeding, and count of the nodes in the lymph system, whereas impediments postsurgery, period of recovery, and patient compliance were secondary observations. 3D printing technology greatly reduced the overall operation time and improved the number of nodes of the lymphatic system. The postoperative prognosis was also improved and the cost of the procedure was reduced to a considerable extent. Thus, researchers concluded that the technology improvises accessibility of surgery and further reduces the cost and complications bearing with it (82).

Another investigation labelled the usage of 3D printing to provide sarcoma patients with favorable performance. The mainstay of therapy was surgical resection, which was difficult to manage. The technique allowed the creation of 3D modelled prostheses, which helped to overcome the limitations of traditional therapy by expressing the body’s 3D anatomical structure. For the users with primary bone sarcoma, the electron beam melting method was employed to simulate the bone structure. The titanium metal was implanted in 23 people at random. All patients were alive after 2-year follow-up period, and implants were evaluated for oncological, mechanical, and infectious problems. Despite the expensive technology, and time-consuming preoperative preparation, ongoing progress will make this technology more user-friendly, with the possibility of robotic surgery in the future. The findings demonstrated the improved resection accuracy for a specific implantation in pelvic surgery (83).

### Drug Delivery

A greater knowledge about the methods to deliver a pharmacological substance to generate a therapeutic effect has resulted from several investigations. Other substance developments have resulted in a slew of novel tools, ideas, and strategies to attain the expected drug delivery. 3D printing, as compared to conventional processes, offers significant benefits in generating extremely complex and personalized models, making it more time and cost-effective (84). The versatility of 3D printing allows the in-house and on-demand creation of customized pharmaceuticals. The most significant approaches for the production of the novel drug delivery system are HME and FDM 3D printing (85, 86). Majority of the studies employed 3D printing for personalized medicine in the form of pellets (87), pill (88), tablet (89), capsule (90), and buccal thin film (91). The privilege of this technology is mostly shown on the solid dosage form.

Liang *et al*. (92) proposed the first-in-human research of a wearable individualized mouthguard as an oral delivery device. The device’s configurable design and variable release rates were achieved via the use of 3D printing (fused deposition process). Clobetasol propionate and polylactic acid and polyvinyl alcohol (polylactic acid and polyvinyl alcohol) were chosen as a model drug and thermoplastic polymers, respectively. Scientists created three sorts of mouthguards using these two polymers, each with its own design and material composition. Human volunteers were used to examine the modelled drug device system’s additional flexibility. According to *in vitro* dissolution investigation, the model drug was released in a steady fashion over a 2-week period. Thermogravimetric analysis was used to determine that these filaments were appropriate for FDM printing due to their great heat stability. In relation to existing manufacturing procedures for regular mouthguards, this 3D printing fabrication approach offered significant time and efficiency benefits, allowing for the production of these devices for immediate use. 3D printing’s enormous potential for the creation and implementation of next-generation therapeutic delivery systems for personalized therapy is skyrocketing (92).

Dumpa *et al*. (93) conducted a unique study to design the gastro-retentive floating pulsatile drug delivery system by loading theophylline as the model drug. For this work, hot-melt extrusion was combined with a fused deposition modelling approach, followed by direct compression. The biopolymers used were hydroxypropyl cellulose (HPC) and ethyl cellulose (EC). The goal of the designed model device was to accomplish dual action by combining floating and pulsatile release after a predetermined lag period in order to improve asthma therapy and wellbeing. Thermal behavior, rigidity assessment, physical attributes, floating and refloating potential, and *in vitro* dissolution rate were all undertaken on the device. Depending on the demands, lag time might be customized (30 min–6 h). The suggested floating pulsatile device showed great potential for delivering pharmaceuticals that needed a long period in the stomach and theophylline pulsatile release. This method decreased unfavorable side effects while increasing patient compliance. In the near future, *in vivo* investigations could be enhanced further (93).

Yan *et al*. (94) tailored oro-dispersible films (ODF) to increase patient acceptance of medication delivery. The drug and excipient were chosen to be levocetirizine hydrochloride and hydroxypropyl methyl cellulose. Plasticizers and fillers were also used to aid disintegration and increase durability. This configurable film was produced using a semi-solid extrusive 3D printing process. Different dosage strengths were indicated on the dosing forms. The developed formulation was also subjected to a series of tests, including drug content, dosage accuracy, tensile properties, contact angle, and drug release determination. 3D printing appeared to be a useful approach for producing personalized ODFs, with acceptable dosage accuracy and a strong linear connection between theoretical model volume and drug content. The flexibility and wetting behavior of 3D-printed ODFs demonstrated that they might be used as an immediate medication delivery device. All ODFs dissolved fully in 2 min in a drug dissolving test, indicating excellent and quick *in vitro* drug release. The high applicability of 3D printing in the creation of uniform ODFs revealed further opportunities in hospitals for a temporary personalized medication delivery system (94).

#### Drug Screening and Drug Modelling

From the pivot plant scale to market, medication development takes a long time and requires huge capital. This puts a strain on the final product that is delivered to the customer. Despite the fact that major ongoing efforts have been made to increase drug efficiency while simultaneously shortening the time it takes for a medicine to reach the market. 3D printing and high-throughput techniques not only enhanced the product model, but also reduced the time of manufacturing, production cost, and compliance (95). Bioprinting organizations have introduced 3D bioprinted tissue models for high-throughput drug testing (96, 97). This platform provides an application to screen out the biomolecules at the early stage of development by providing *in vitro* tissue model. This technology leads to bypassing the trial and error failures and simultaneously saving time and money in the development process. Detailed knowledge with a large overview was given in many earlier publications (98, 99). Conventional 2D cell culture studies and animal models were used to elucidate the underlying cellular and molecular pathways that underlie in a variety of human illnesses, but they had a number of disadvantages. Through the integration of *in vitro* 3D cell culture models with cell lines, different breakthroughs such as microfluidic devices, tissue-on-a-chip, and organ-on-a-chip have been launched for recapitulation of the biological properties and functions of native human tissues, organs, and circulation (100, 101).

#### Wound Healing

Wound dressing is a therapy that involves reducing the infection and aiding wound healing. Other complicated reactions may be triggered by this classic way. As a result, 3D printing delivers a patient-specific antibacterial impact, as well as additional benefits such as the capacity to change wound dressing’s dimensional qualities, easy medication loading, use of a wide range of materials, and oxygen penetration due to pore design (102).

In the realm of wound dressing, Ilhan *et al*. (103) surmounted a significant obstacle. Acute wounds were a severe health danger that can be managed by 3D printing. Satureja cuneifolia plant extract (SC) was used as a model in this study, and it was found to be effective in treating diabetic ulcers. The extract was mixed with various polymers, such as sodium alginate and polyethylene glycol, in varying ratios. For morphological and chemical characterization, SEM and FT-IR measurements were employed. Along with the dissolution studies, mechanical and thermal properties, swelling, and degradation behaviors were also investigated. Antimicrobial activity was confirmed against a few types of topical bacteria. MTT assay was used to test the biocompatibility, cell viability, and cytotoxicity of the generated 3D-printed composite scaffolds with varying quantities of medicinal plant concentrations. The results were similar to those of the ampicillin antibiotic, which was used as a control in the wound healing process. This demonstrated that the model was a good fit for the wound healing created by 3D printing (103).

### Miscellaneous

#### COVID Essentials

COVID-19, a worldwide outbreak of the spread of the virus mainly affected the respiratory passage. The limited supply of N95 masks, personal protective kits, face shields, testing kits, etc. were a great backstep for controlling the growth of spread among people. In the pandemic, 3D printing technology gave a way for the fabrication of these materials at a rapid rate and efficient manner to meet the needs of physicians, needy ones, and patients (104). Polymers like polyester, polycarbonate, and polyvinyl chloride are used in 3D printing for the modelling of face shields. 3D laser scanning is employed in the development of N95 masks. Material extrusion technique is applied to thermoplastic polymers including styrene complex for the same.

Medications which are generally used for COVID control such as lopinavir/ritonavir, chloroquine, and hydroxychloroquine pills were also fabricated at the higher rate of layer-by-layer deposition of drugs by fused filament, ink jet, and powder extrusion (105, 106).

#### Cosmeceuticals

Cosmetic medicine is stepping up the importance among the population in curing or preventing many visual informalities. The increased surge requires a lot of advances and implementation of the technology in this field. Therefore, 3D printing promotes the enhancement of this technology with further added benefits for the patients. Mostly, UV rays from the sun lead to many skin-related constraints and are also harmful to the environment. Some percentage of the rays penetrates the normal aperture of human skin leading to generation of the reactive oxygen species, alteration of the gene, DNA and cell damage, and in worst cases, causes skin cancers (107). The most conventional strategy for the avoidance of consequences is the reduction of exposure to rays or the use of sunscreens which generally restrict the passage into the skin layers. The following study material expresses the application of 3D printing in the cosmeceutical field for further reference for the reader (108).

#### Stem Cell

Feng *et al*. (109) utilized stem cells in the fabrication procedure. The robustness and cost-effectiveness of the models were enhanced by real-time monitoring of the 3D approach. A flexible technique for producing scaffolds from alginate and gelatin was established. A comparison of the model and hydrogel forms of the identical polymers was done. The produced model had a better pattern and had high tensile strength, according to the parameters.

HepaRG cells and embryonic stem cells (ESCs) were examined for a particular architecture and cell proliferation potential as a result of this culture. The pluripotent cells increased hepatic differentiation and proliferation which were validated. The scaffold was cross-linked and de-cross-linked to safely remove it from the structure. This research outlined an innovative and adaptable method for creating 3D printing scaffolds for large-scale cell multiplication and safe collection for use in a variety of downstream applications (109).

#### Veterinary Medicine

As aforementioned, 3D printing gained a milestone in many of the pharmaceutical sector and also promotes surgical applications. Hence, the benefits are not restricted only to human use; it can be extended to veterinary purposes. In different research studies, the various stretch in animal science has already been evaluated. It is very much helpful in aiding the surgery and imaging, so as to easy diagnosis and reducing the errors to improve the safety of animals (110, 111). Kim *et al*. (112) adopted 3D printing to recreate a bone model scaffold following tumor excision surgery. In order to restore maxillofacial abnormalities, tissue functions must be retained in addition to anatomical consistency and appearance, and 3D printing may be able to meet these demands. The problem in the female dog was reported using the required imaging technique, and an adapted surgery plan was established to treat the malignancy. The addition of a scaffolding framework comprised of polymers such as polycaprolactone and betatracalciumphosphate. The 3D model was added after the procedure, and further follow-up investigations were conducted on it. In the periodic CT scans and oral examinations, the outcome was successful without any specific complications. This was thought to be the first instance of maxillary bone defect repair utilizing a 3D-printed scaffold for animal use (112). Furthermore, additional applications employing 3D printing are enlisted in Table III.

**Table III:** 3D Printing Applications from Bibliographic Corpus

Applications	Product	Comments	Ref
Drug delivery	Indomethacin loaded transdermal patches	• Patches were modelled with inkjet technology • Drug release and permeability were enhanced in the modelled patches.	(113)
	Ketoprofen & lidocaine hydrochloride buccal films	• Films were fabricated using the combination of FDM and inkjet printing. • Films showed lack of toxicity and improved mucoadhesive properties.	(114)
	Caffeine incorporated tablet	• Fabricated by employing binder jet technique. • Hydroxypropyl cellulose was selected as the suitable binder.	(115)
	Aripiprazole loaded orodispersible film	• Films were modelled using FDM. • Polyvinyl alcohol was concluded as suitable polymer. • Dispersible films with desired characteristics were developed.	(116)
	Pellets bearing paracetamol and ibuprofen	• Method of development was selective laser sintering. •Ethyl cellulose containing printlets expressed prolonged drug release.	(117)
	Hydrogels containing ibuprofen	• Fabrication was done by stereolithography. • Hydrogel with high water content released drug at faster rate.	(118)
	Insulin capped microneedles	• Stereolithography was the modelling technique. • Pyramid and cone-shaped microneedles were further coated by inkjet printing. • Microneedles expressed desired delivery insulin	(119)
	Lidocaine & piroxicam scaffold	• Modelled by inkjet printing technique. • Good entrapment, and solidification of the drug was observed.	(120)
Medical devices	Cellular scaffolds for orthopedic implants	• Modelled by selective laser melting and selective electron beam melting • Titanium-aluminum alloy model was considered to be optimum material in implant fabrication.	(121)
	Anthropomorphic thorax phantom	• Used for testing and optimization of imaging devices. • Gypsum, nylon, and silicone were employed to mimic the human lung. • Phantom model closely resembled the patient’s lungs. • Finds its use in cancer diagnostics.	(122)
	Multidrug loaded hearing aids	• Ciprofloxacin and fluocinolone acetonide were the model drugs. • Digital light processing 3D printing was used to generate hearing aids. • The devices exhibited prolonged release for a period of 2 weeks.	(123)

## 4D Printing

Despite the fact that 3D printing aids in the production of many complicated structures, microstructures generated by 3D printing are static. As a result, 4D printing creates a complex structure that changes over time and responds to environmental stimuli in the desired way. The scaffolded model is no longer static, and it may be converted into complex structures by altering the size, shape, property, and functioning of the model in response to external inputs (124). Unlike 3D printing, this technique is printer-independent and is time-bound. The design of 4D-printed structures should be meticulously preprogrammed, taking into consideration any expected time-dependent component deformations. Smart materials, which may become more extensible, malleable, or deformable in reaction to applied stimuli, are another important feature of 4D printing technology (125).

## Challenges for Implementing 3D Printing

Though 3D printing seems possible advantageous in every field of health care medicine, the restrictions are yet obvious due to certain aspects. Few challenging domains include safety concerns, regulatory perspective, fabrication materials, technological difficulties, and anti-counterfeiting (126).

Safety is the greatest concern during drug manufacturing. During the 3D printing fabrication method, the smaller airborne particles may result which leads to certain respiratory problems to the manufacturers. The regulatory perspective of any formulation or medical device is also a major step to leap. FDA published “Technical Considerations for Additive Manufacturing of Medical Devices” in 2017 to offer significant regulative suggestions, and prerequisites for the authorization of three-dimensional pharmaceutical materials and biomedical devices (127). As a result of this, many medical devices found its way into the market by facing challenges. But the only pharmaceutical product approved by the regulatory body was Spritam. Moreover, the fact is still unclear whether the guidelines fit all processes and parameters or the final product alone.

Fabricating materials should also comply with the regulatory guidelines. Despite the fact that bioinks must have desirability with drugs and other excipients of formulation, it is also compatible with the human body. Bioink’s safety is also considered to an extent to defend it from generating any harmful toxic substance throughout the shelf-life (128). Due to losing framework of guidelines, many counterfeit devices with improper quality and safety attributes find its place in the market. These fake and unauthorized replicas of indigenous products are often misleading patients with substandard devices. An additional issue is that cellular architectures and components change among patients, rendering it unfeasible to sustain or reproduce *in vivo* settings, resulting in delayed advancement. The barrier between laboratory and clinical uses of 3D bioprinting is widening due to challenges with selecting appropriate types of technology, cells, and construction material for fabrication (129, 130).

## Conclusion

Additive manufacturing is extending beyond its traditional function, and is now used in a wide range of sectors ranging from lightweight engineering to energy technologies, medicine, and far more. 3D bioprinting is one of the most potential technologies for producing cell-loaded scaffolds which could further differentiate and proliferate. The rise of personalized implants and multiactive medicine is a great leap in technology. The patient-specific medicines cut short many harmful sides of the conventional dosage forms. Biopolymers involved in the fabrication also proved to improve the drug efficiency and potentiation. Albeit the printers and biomaterials used are exorbitant, technology is improving steadily, and prices will drop to the point where it will be ideal for even large-scale production. This breakthrough provides increased advantages to medical students, practitioners, and patients. With the rising use of implants and prostheses for a diverse population, the quality of life is improved in comparison to conventional treatment procedures. We can expect the greater stable technology with further more improved characteristics in the pharma culture. The cell lines incorporated should be mechanically stable over the period with adaptable biological character still being a hurdle for a full stretch. The functionality and characteristics of bioprinted tissue-like constructions may be evaluated using machine learning. With the advancement and maturation of 3D bioprinting, deep learning is expected to play a large role in improving the process and product quality.
